# EDITORIAL: Stroke—Pathophysiology and New Therapeutic Strategies

**DOI:** 10.3390/biomedicines10071529

**Published:** 2022-06-28

**Authors:** Roger E. Kelley

**Affiliations:** Department of Neurology, Ochsner/LSU Health Sciences Center, Shreveport, LA 71130, USA; roger.kelley@lsuhs.edu

This Special Issue of *Biomedicines* highlights recent advances in stroke evaluation and management and provides some pertinent information about potential new directions of stroke intervention in the research realm. The duration of my experience in stroke assessment allows me to try to put things into perspective in terms of the dramatic advances in stroke care. For example, it was in the 1970s that the CT brain scan became available. This was a monumental contribution to the ability to diagnose a stroke. It allowed the ready differentiation of ischemic versus hemorrhagic stroke and helped to exclude alternative explanations for a patient presenting an acute neurological deficit. In the 1980s, the MRI brain scan became available and evolved from T1-weighted, T2-weighted, and proton density sequences to FLAIR sequences and diffusion-weighted images (DWI). DWI generally allows ready identification of an acute ischemic stroke based upon hyperintensity of the involved cerebral region compared to the correlative hypointensity in the apparent diffusion coefficient (ADC) sequence. The presence of DWI hyperintensity without a correlative FLAIR hyperintensity is now used to identify salvageable ischemic tissue in a so-called “wake-up” stroke. Prior to 1995, the major issue confronting a neurologist evaluating a patient with acute ischemic stroke was whether or not to use intravenous heparin. There were various presentations to consider, such as “stroke-in-evolution” or “crescendo” TIA, with an ongoing debate about the potential merits versus the risks of such intervention. However, enthusiasm for acute heparin use was tempered by the TOAST study [1]. It was in 1995 that the intravenous tissue plasminogen activator (TPA) came to the forefront of acute ischemic stroke management [2] for those presenting within the time window without a clearly defined contraindication or refusal of this agent by the patient or the family. Tenecteplase appears to be making inroads as an alternative thrombolytic agent. In 2015, with MR CLEAN [3] and a plethora of additional further supportive studies, thrombectomy was introduced to the accepted management of acute ischemic stroke secondary to large vessel occlusion (LVO). In selected patients, this procedure has the potential to markedly improve the functional outcome. The efficacy of this intervention correlated with advances in neuroimaging by combining a CT brain scan with CTA and CT perfusion or alternative MRI modalities. The previous, somewhat nihilistic approach toward acute stroke was replaced by the development of stroke units, fellowship-trained vascular neurologists, fellowship-trained neuro-interventionalists, as well as primary and comprehensive stroke centers with a 24/7 “Stroke Team” to promote rapid access to these interventions with the potential for improved outcomes. I recall being cautioned about entering the field of neurology because “nothing could be done” in terms of helping various patients seen by the neurology service. Thankfully, this is no longer the case, and this issue provides a valuable summary of where we presently stand in this exciting and evolving process.
